# Tetrandrine inhibits the proliferation of mesangial cells induced by enzymatically deglycosylated human IgA1 via IgA receptor/MAPK/NF-κB signaling pathway

**DOI:** 10.3389/fphar.2023.1150829

**Published:** 2023-06-15

**Authors:** Wencheng Xu, Wanci Song, Shuhe Chen, Shanshan Jin, Xue Xue, Jinwen Min, Xiaoqin Wang, Pengtao You

**Affiliations:** ^1^ Department of Pharmacy, Hubei Provincial Hospital of Traditional Chinese Medicine, Wuhan, China; ^2^ Hubei Key Laboratory of Theory and Application Research of Liver and Kidney in Traditional Chinese Medicine, Affiliated Hospital of Hubei University of Chinese Medicine, Wuhan, China; ^3^ Hubei Province Academy of Traditional Chinese Medicine, Wuhan, China; ^4^ Hubei Key Laboratory of Resources and Chemistry of Chinese Medicine, Hubei University of Chinese Medicine, Wuhan, China; ^5^ Department of Nephrology, Hubei Provincial Hospital of Traditional Chinese Medicine, Wuhan, China; ^6^ The First Clinical Medical College, Jinzhou Medical University, Jinzhou, China

**Keywords:** tetrandrine, mesangial cells, IgA nephropathy, IgA receptor, proliferation

## Abstract

**Objective:** Despite the use of renin-angiotensin system blockade and immunosuppressive drugs, including corticosteroids, the current treatment regimens for Immunoglobulins A nephropathy (IgAN) are severely limited. The proliferation of mesangial cell and deposition of deglycosylated human IgA1 immune complex are the most common pathologic features of IgAN. We examined the tetrandrine potential of suppressing the proliferation of mesangial cells and explored its underlying mechanisms with a focus on IgA receptor/MAPK/NF-κB signaling pathway.

**Methods:** Standard human IgA (native IgA) were enzymatically desialylated (deS IgA) or further degalactosylated (deS/deGal IgA) using neuraminidase and *β*-galactosidase. Rat glomerular mesangial cells (HBZY-1) and human renal mesangial cells (HRMC) stimulated by IgA were used to observe the suppressive effect of tetrandrine. The MTT assay was used to detect the cell viability. The protein expression of IgA receptor/MAPK/NF-κB signaling pathway was examined by Western blot. Cell cycle analysis was measured by flow cytometer.

**Results:** Native IgA and deS IgA showed limited stimulation effect on both HBZY-1 cells and HRMCs, whereas deS/deGal IgA significantly stimulated the proliferation of both HBZY-1 cells and HRMCs (*p* < 0.05). Compared with non-stimulation of deS/deGal IgA, 1–3 μM of tetrandrine had stronger inhibitory effect on the proliferation of HBZY-1 cells and HRMCs with the stimulation of deS/deGal IgA (*p* < 0.05), suggesting that tetrandrine possibly inhibited the proliferation of mesangial cells induced by deglycosylated human IgA1 specifically. Molecular mechanism study revealed that tetrandrine decreased the expression of IgA1 receptor, CD71 and β4GALT1, and inhibited the activation of MAPK/NF-κB significantly (*p* < 0.05). Moreover, these inhibitory effect of tetrandrine caused cell cycle arrest and stopped the cell growth in the S phase companied with the upregulating of cyclin A2 and downregulating of cyclin D1.

**Conclusion:** Taken together, tetrandrine inhibited the proliferation of mesangial cells induced by enzymatically deglycosylated human IgA1 via IgA receptor/MAPK/NF-κB signaling pathway. Based on these potential molecular mechanisms, tetrandrine would be an appealing therapeutic option for IgAN.

## 1 Introduction

Immunoglobulins A nephropathy (IgAN) is recognized as the most common primary glomerular glomerulonephritis throughout the world. The common clinical manifestation of IgAN patients is asymptomatic hematuria and proteinuria (Zhang et al., 2016; Pattrapornpisut et al., 2021). After a slow progression in 20 years, approximately 30%–40% of IgAN patients were reported to develop end-stage renal failure which had to receive dialysis or kidney transplantation (Zhang et al., 2016).

The histological feature of IgAN is mesangial deposition of the IgA1-containing immune complexes and cell proliferation (Lai et al., 2016). Usually, the structure of human IgA1 has O-glycosylation on the serine and threonine residues in the hinge region of heavy chain. The disaccharide of O-glycan side chains is formed by a primary N-acetylgalactosamine and a secondary β1,3-linked galactose, both of which could be sialylated. However, the hinge region of heavy chain in IgA1 isolated from the serum and the mesangial areas of IgAN patients’ renal biopsy were certified to be aberrantly glycosylated or galactose-deficient (Wang et al., 2016). Moreover, the glomerular injury of IgAN is recognized as predominantly mediated by the activation of mesangial cells which are stimulated by the deposited IgA1 in mesangial areas since IgAN is not associated with a significant glomerular cell infiltrate in most cases (Molyneux et al., 2017). Cross-linking of IgA receptors, such as the transferrin receptor (CD71) and β1,4-galactosyltransferase 1 (β4GALT1), elicits proliferation and a proinflammatory in mesangial cells (Tamouza et al., 2012; Molyneux et al., 2017). The mitogen-activated protein kinase (MAPK) and nuclear transcription factor-κB (NF-κB) signal transduction pathways contribute to the activation and proliferation of mesangial cells induced by galactose-deficient IgA1 (Leung et al., 2008; Tamouza et al., 2012).

Tetrandrine, one of the main active components in Fang Ji (*Stephania tetrandrae Root*), has been approved for the treatment of silicosis and rheumatic arthritis in China (Xu et al., 2017). Recent evidence suggests that tetrandrine could suppress the proliferation of T cells stimulated by concanavalin A through multiple cellular signaling, such as NF-κB, MAPK, and PI3K/Akt/mTOR (Xu et al., 2019b; Xu et al., 2021). Moreover, tetrandrine was confirmed to inhibit the activation and proliferation of mesangial cells induced by aberrantly glycosylated human IgA1 or IL-1β (Wu et al., 2011; Xia et al., 2022). In the present study, we evaluated the tetrandrine potential of suppressing the proliferation of mesangial cells induced by enzymatically deglycosylated human IgA1 molecules and explored its underlying mechanisms with a focus on IgA receptor/MAPK/NF-κB signaling pathway.

## 2 Materials and methods

### 2.1 Reagents and antibodies

Dulbecco’s modified Eagle’s medium (DMEM) and fetal bovine serum (FBS) were obtained from Gibco BRL (Grand Island, NY, United States). Tetrandrine (purity: more than 98%) was provided by Chengdu Must Bio-Technology Co., Ltd. EMEM medium was purchased from BeNa Culture Collection (Xinyang, China). National standard human IgA was provided by National Institutes for Food and Drug Control. Neuraminidase and *β*-galactosidase were all purchased from Shanghai Yuanye Biotechnology Co., Ltd. Primary antibodies against ERK1/2 (dilution 1:1000, #4695), p-ERK1/2 (dilution 1:1000, #4370), JNK (dilution 1:1000, #9258), p-JNK (dilution 1:1000, #4668), p38 (dilution 1:1000, #8690), p-p38 (dilution 1:1000, #4511), NF-κB (dilution 1:1000, #8242), Cyclin D1 (dilution 1:1000, #55506), Cyclin B1 (dilution 1:1000, #12231), Cyclin A2 (dilution 1:1000, #4656) and CD71 (dilution 1:1000, #13113) antibodies were provided by Cell Signaling Technology. Anti β4GALT1 (dilution 1:500, #abs148346) antibody was purchased from Absin. *β*-actin (dilution 1:5000, #66009-1-Ig) antibody was provided by Proteintech Group. All other reagents are the highest quality reagents provided by commercial suppliers.

### 2.2 Cell cultures

Rat glomerular mesangial cells (HBZY-1, #4201RAT-CCTCC00124) were obtained from China Center for Type Culture Collection (Wuhan, China). HBZY-1 cells were routinely grown in DMEM medium supplemented with 10% FBS and 1% penicillin/streptomycin and cultured at 37°C in a humidified atmosphere of 5% CO_2_ as described before (Li et al., 2022). Human renal mesangial cells (HRMC, #BNCC341276) were provided by BeNa Culture Collection (Xinyang, China). HRMC culture followed with the instructions of manufacturer. Cells between passages three and seven were employed in the present study.

### 2.3 Deglycosylation of human IgA1

Standard human IgA was enzymatically digested with neuraminidase and *β*-galactosidase according to the following procedure. Standard human IgA was divided into three groups of 15 mg each. The first group was dissolved in 15 mL of 10 mM/L sodium acetate buffer (SAB, pH 5.0) without any enzymatic process, named as native IgA. The second group was dissolved in SAB, and 7.5 mL of neuraminidase (20 mU/mL) was added to remove N-acetylneuraminic acid from the IgA protein, named as deS IgA. The third group was dissolved in SAB, and 7.5 mL of neuraminidase (20 mU/mL) and 7.5 mL of *β*-galactosidase (20 mU/mL) were added to remove N-acetylneuraminic acid and galactose together, nameds as deS/deGal IgA. All samples were incubated overnight at 37°C as described before (Tomana et al., 1999; Sano et al., 2002).

### 2.4 MTT assay

The 3-(4,5-dimethylthiazol-2-yl)-2,5-diphenyl tetrazolium bromide (MTT, Sigma, MO, United States) assay was used to detect the cell viability. After cell treatment, 10 µL of 5 mg/mL MTT was added to each well with a 4 h-incubation at 37°C. Then, the plates were centrifuged at 375 × g for 5 min followed by discarding the supernatant. Next, precipitated formazan crystals produced by growing cells was dissolved in 150 µL of dimethyl sulfoxide in each well followed by shaking 10 min. Finally, the absorbance was measured at 450 nm by Spark 10 M microplate reader (Tecan, Männedorf, Switzerland). The IC_50_ values of tetrandrine were calculated from dose-response curves as we described before (Xu et al., 2019a).

### 2.5 Cell cycle analysis

HBZY-1 cells were seeded in 6-well plates and incubated with DMEM medium and stimulated by 1 mg/mL of deS/deGal IgA. Then, the cells were treated by blank solvent, 1, 2.5 and 3 µM of tetrandrine respectively. After 48 h, the cells were harvested and fixed by the treatment with 70% ethanol at 4°C for 1 h. Propidium iodide (100 μg/mL,#P4170, Sigma Chemical Co.) and RNaseA (1 mg/mL,#R5500, Sigma Chemical Co.) were added to stain cellular DNA at 37°C and in dark for 30 min. Finally, the content of cellular DNA was examined by flow cytometry (FACSCalibur, BD Bioscience, CA, US). MODFIT LT™ (V3.1, Verity Software House, Topsham, ME, United States) was used to analyze the data as we described before (Xu et al., 2019b).

### 2.6 Western blot

After harvest, cells were treated by RIPA lysis kit combined with the protease inhibitor (Beyotime). Protein concentration of cell lysate was examined by BCA protein assay kit (Takara, Kyoto, Japan). The cell extracts were subsequently separated by SDS-polyacrylamide gel electrophoresis and then transferred to nitrocellulose membranes (Millipore, MA, United States). After blocking in 5% nonfat milk at room temperature for 2 h, membranes were incubated with primary antibodies overnight at 4°C, following with the treatment by horseradish peroxidase (HRP) coupled secondary antibody (#7076, Cell Signaling Technology) for 1 h at room temperature. Finally, membranes were treated by ECL reagent (Bio-Rad, CA, United States), signals were detected by FluorChem FC3 system (ProteinSimple, CA, United States) and then quantitatively analyzed by ImageJ software as we described before (Xu et al., 2019b).

### 2.7 Statistical analysis

Differences in the percentages of viable cells, percentage of cells in cell-cycle phases and the expressions of proteins were examined by Bonferroni Multiple Comparison Tests. These above analyses were performed by using GraphPad PRISM 9.5 (GraphPad Software Inc., San Diego, CA). In each case, two-sided *p* values < 0.05 were considered to be significant.

## 3 Results

### 3.1 Effects of tetrandrine on the proliferation of HBZY-1 cells

**FIGURE 1:**
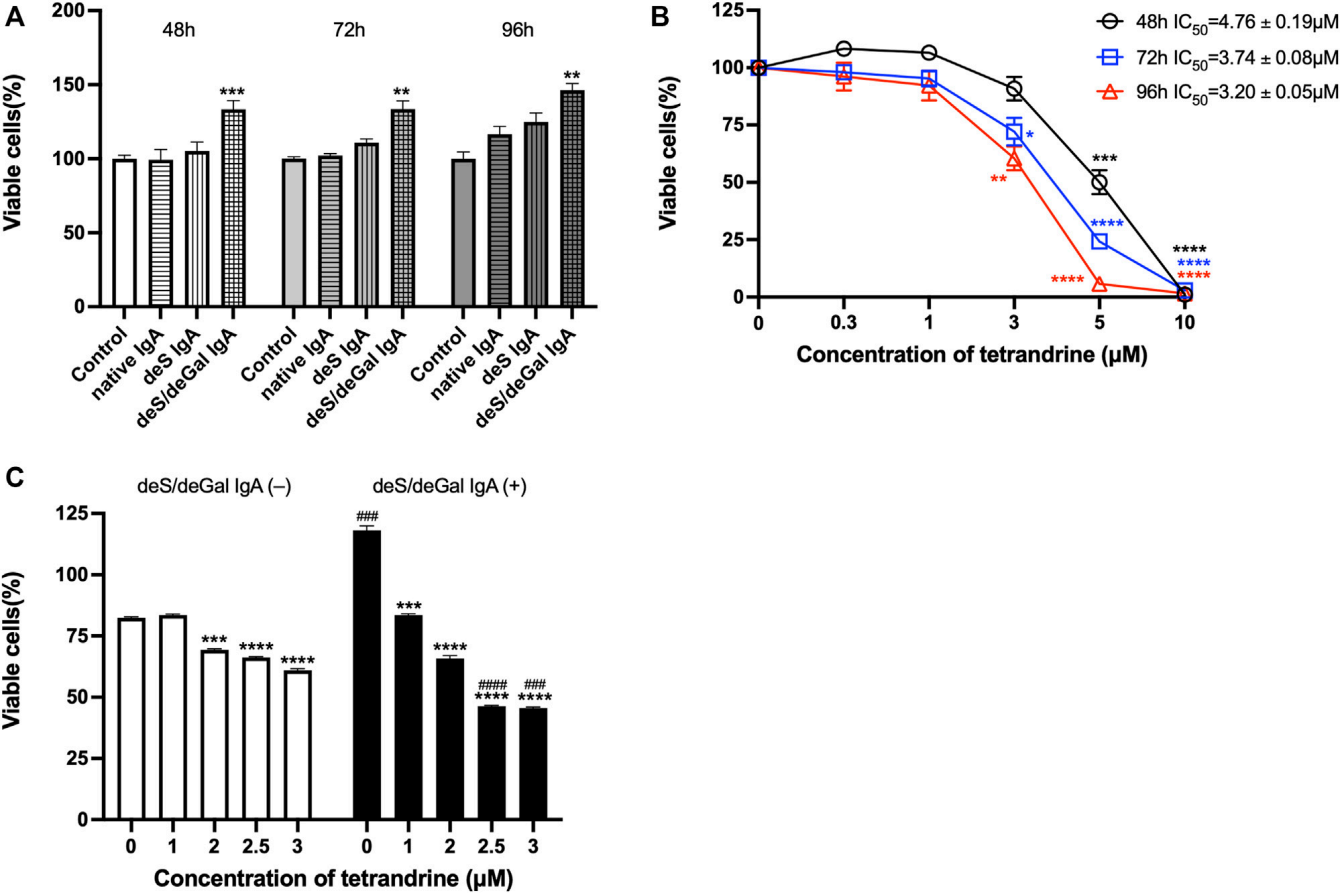
Effects of tetrandrine on the proliferation of HBZY-1 cells. **(A)** The stimulation effects of enzymatically deglycosylated human IgA1 on cell proliferation of HBZY-1 cells were examined. Cells were co-cultured with blank solvent, native IgA, deS IgA and deS/deGal IgA for 48, 72, and 96 h respectively. **(B)** HBZY-1 cells were stimulated by deS/deGal IgA and treated with different concentrations of tetrandrine (0, 0.3, 1, 3, 5 and 10 μM) for 48, 72, and 96 h respectively. IC_50_ values of tetrandrine were calculated by GraphPad PRISM 9.5. **(C)** HBZY-1 cells were treated with different concentrations of tetrandrine (0, 1, 2, 2.5 and 3 μM) in the presence or absence of deS/deGal IgA for 48 h. Cell proliferation was determined by MTT assay. The data were expressed as means ± S.E.M. Statistical analyses were performed using Bonferroni’s multiple comparison tests, *p < 0.05, **p < 0.01, ***p < 0.001 and ****p < 0.0001, as compared to the group treated by blank solvent, ^###^ *p* < 0.001 and ^####^ *p* < 0.0001, as compared to the group in the absence of deS/deGal IgA, respectively. (*n* = 6).

We first examined the stimulation effects of deS/deGal IgA on cell proliferation of HBZY-1 cells. Cells were co-cultured with blank solvent, native IgA, deS IgA and deS/deGal IgA for 48, 72, and 96 h respectively. As shown in Figure 1A, only deS/deGal IgA significantly stimulated the proliferation of HBZY-1 cells (*p* < 0.01). Next, we observed the suppressive effects of tetrandrine on the proliferation of HBZY-1 cells induced by deS/deGal IgA. As shown in Figure 1B, tetrandrine inhibited the proliferation of HBZY-1 cells stimulated by deS/deGal IgA in a dose- and time-dependent manner. Specifically, 1 μM of tetrandrine significantly inhibited the proliferation of HBZY-1 cells stimulated by deS/deGal IgA with little cytotoxic effect on HBZY-1 cells alone (*p* < 0.001, Figure 1C). Compared with non-stimulation of deS/deGal IgA, 2.5 and 3 μM of tetrandrine showed stronger inhibitory effect on the proliferation of HBZY-1 cells with the stimulation of deS/deGal IgA (*p* < 0.001, Figure 1C).

### 3.2 Effects of tetrandrine on the expression of IgA receptor in HBZY-1 cells

**FIGURE 2:**
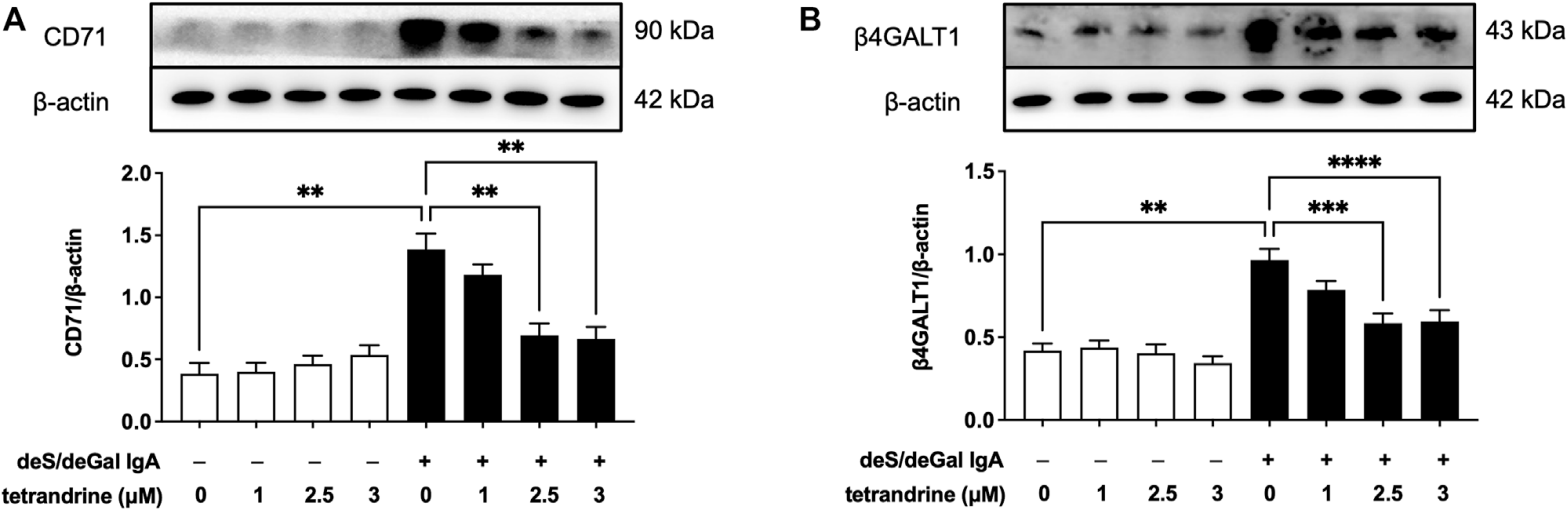
Effects of tetrandrine on the expression levels of IgA receptor protein in HBZY-1 cells. Cells were treated with different concentrations of TET (0, 1, 2.5 and 3 μM) in the presence or absence of deS/deGal IgA for 48 h. The cell lysates were examined by Western blot. More than three independent experiments were carried out and representative results were shown in the figures. *β*-actin was used as internal control. Statistical analyses were performed using Bonferroni’s multiple comparison tests, **p < 0.01, ***p < 0.001 and ****p < 0.0001 respectively. (*n* = 6).

To investigate the possible molecular mechanism for the inhibitory effect of tetrandrine against the stimulation of deS/deGal IgA, effects of tetrandrine on the expression of IgA receptor in HBZY-1 cells were examined. Treatment of deS/deGal IgA significantly stimulated the expression of CD71 and β4GALT1 (*p* < 0.01, Figure 2). 1–3 μM of tetrandrine inhibited the expression of both CD71 and β4GALT1 significantly and dose-dependently (*p* < 0.01, Figure 2).

### 3.3 Effects of tetrandrine on MAPK/NF-κB signaling pathway in HBZY-1 cells

**FIGURE 3:**
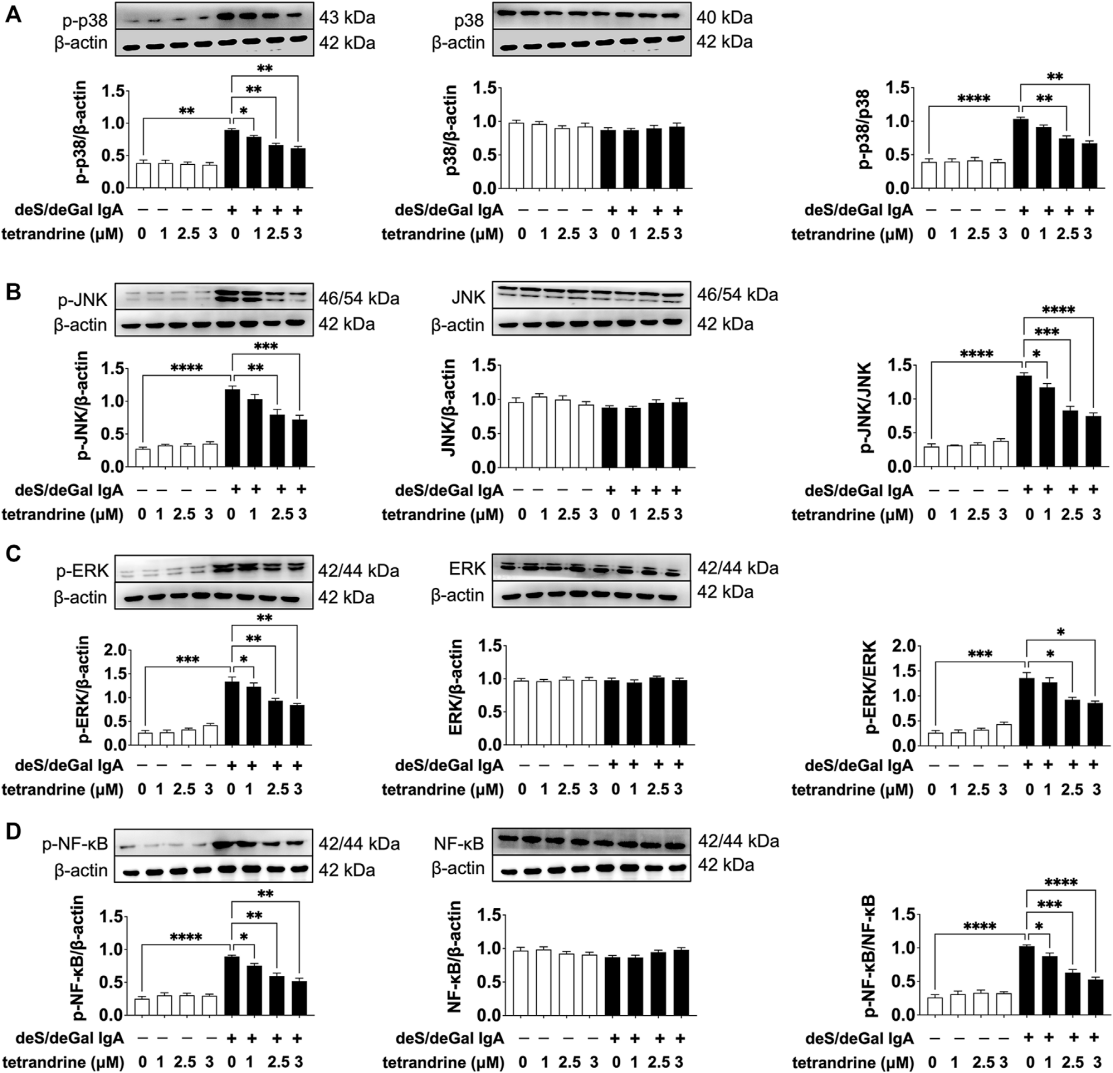
Effects of tetrandrine on the expression levels of MAPK/NF-κB in HBZY-1 cells. Cells were treated with different concentrations of TET (0, 1, 2.5 and 3 μM) in the presence or absence of deS/deGal IgA for 48 h. The cell lysates were examined by Western blot. More than three independent experiments were carried out and representative results were shown in the figures. *β*-actin was used as internal control and re-used to compare the expression levels of MAPK/NF-κB and their phosphorylation protein conveniently. Statistical analyses were performed using Bonferroni’s multiple comparison tests, *p < 0.05, **p < 0.01, ***p < 0.001 and ****p < 0.0001 respectively. (*n* = 6).

MAPK/NF-κB signaling pathway maintains cell proliferation. To investigate the possible mechanism of tetrandrine’s inhibitory effect, we examined the effects of tetrandrine on MAPK/NF-κB activation in HBZY-1 cells. As shown in Figure 3, treatment of deS/deGal IgA significantly stimulated the phosphorylation of p38, JNK, ERK and NF-κB (*p* < 0.01). 1–3 μM of tetrandrine decreased the expression of p-p38, p-JNK, p-ERK and p-NF-κB in a dose-dependent manner with little effect on the expression of p38, JNK, ERK and NF-κB (*p* < 0.05, Figure 3).

### 3.4 Effects of tetrandrine on cell cycle arrest in HBZY-1 cells

**FIGURE 4:**
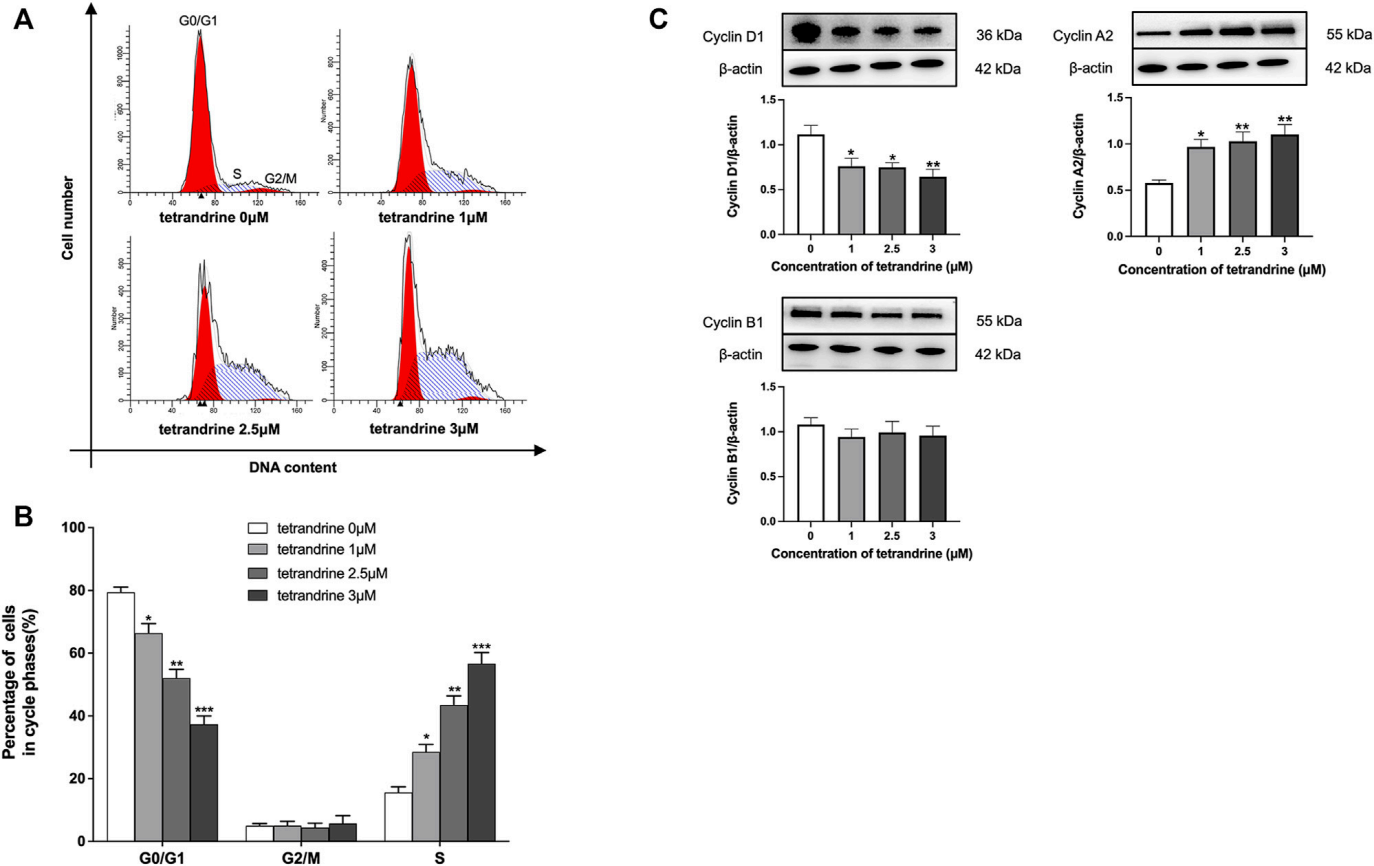
Tetrandrine triggers cell cycle in HBZY-1 cells. Cells stimulated by deS/deGal IgA were treated with different concentrations of tetrandrine (0, 1, 2.5 and 3 μM) for 48 h. **(A)** Cell cycle was analyzed by flow cytometer. The peaks marked in the figure represent G0/G1, S and G2/M phases in the cell cycle, respectively. **(B)** Percentages of cell numbers in the cell cycle after 48 h of the agent treatment were. **(C)** The cell lysates were examined by Western blot. *β*-actin was used as internal control. More than three independent experiments were carried out and representative results were shown in the figures. Statistical analyses were performed using Bonferroni’s multiple comparison tests, *p < 0.05, **p < 0.01, and ***p < 0.001 as compared to the group treated by blank solvent respectively. (*n* = 6).

To examine the effects of tetrandrine on cell cycle arrest in HBZY-1 cells, HBZY-1 cells were stimulated by deS/deGal IgA and treated with 0, 1, 2.5 and 3 μM of tetrandrine respectively. As shown in Figures 4A, B, tetrandrine significantly caused cell cycle arrest and stopped the cell growth in the S phase in a dose-dependent manner (*p* < 0.05). The treatment of tetrandrine also decreased the percentage of cells at G0/G1 phase dose-dependently and significantly (*p* < 0.05). 1–3 μM of tetrandrine showed limited influence on the population of cells at G2/M phase. Accordingly, 1–3 μM of tetrandrine inhibited the expression of cyclin D1 but enhanced the expression of cyclin A2 significantly and dose-dependently (*p* < 0.05, Figure 4C).

### 3.5 Effects of tetrandrine on the proliferation of HRMCs

**FIGURE 5:**
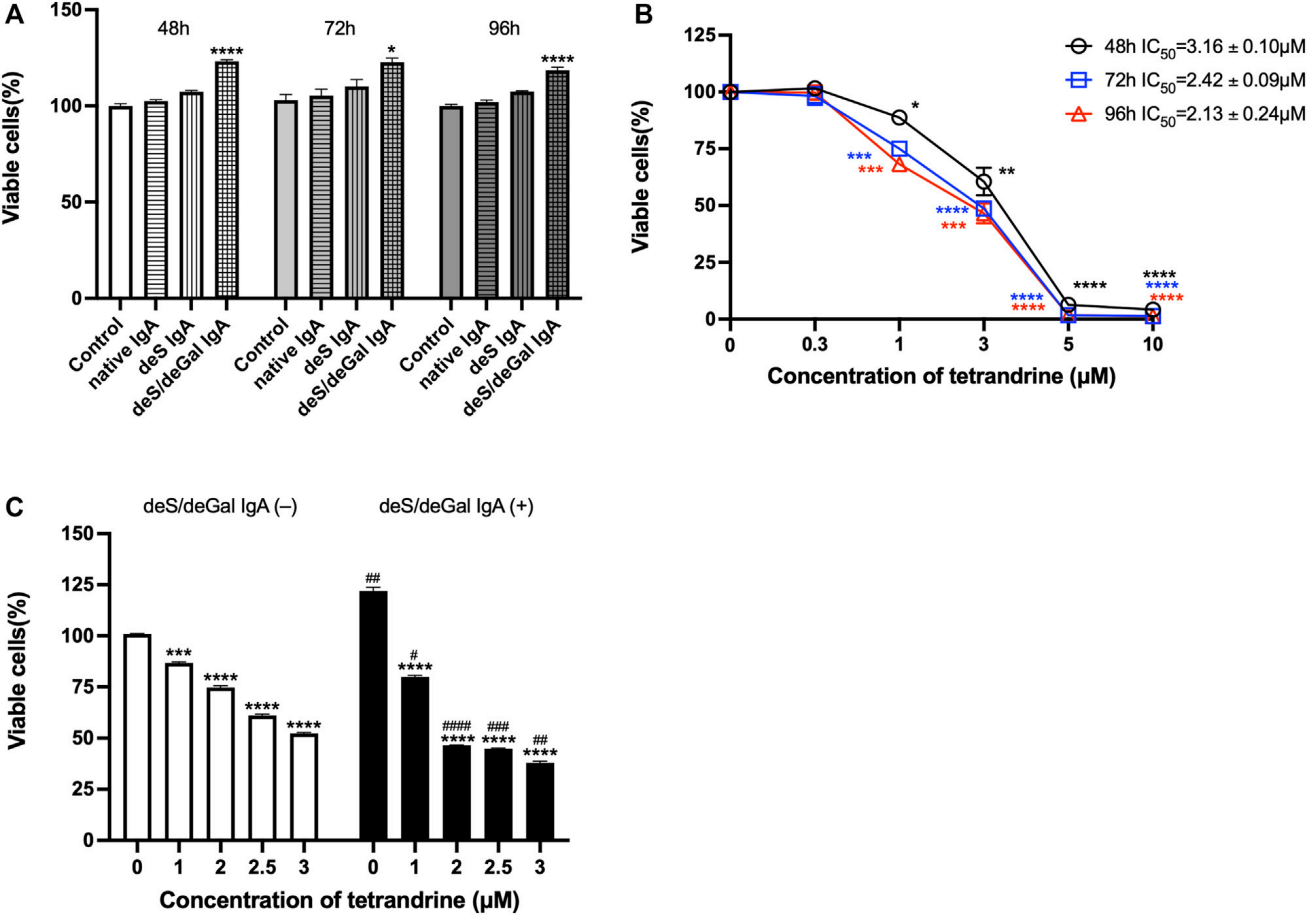
Effects of tetrandrine on the proliferation of HRMCs. **(A)** The stimulation effects of enzymatically deglycosylated human IgA1 on cell proliferation of HRMCs were examined. Cells were co-cultured with blank solvent, native IgA, deS IgA and deS/deGal IgA for 48, 72, and 96 h respectively. **(B)** HRMCs were stimulated by deS/deGal IgA and treated with different concentrations of tetrandrine (0, 0.3, 1, 3, 5 and 10 μM) for 48, 72, and 96 h respectively. IC_50_ values of tetrandrine were calculated by GraphPad PRISM 9.5. **(C)** HRMCs were treated with different concentrations of tetrandrine (0, 1, 2, 2.5 and 3 μM) in the presence or absence of deS/deGal IgA for 48 h. Cell proliferation was determined by MTT assay. The data were expressed as means ± S.E.M. Statistical analyses were performed using Bonferroni’s multiple comparison tests, *p < 0.05, **p < 0.01, ***p < 0.001 and ****p < 0.0001, as compared to the group treated by blank solvent, ^#^ *p* < 0.05, ^##^ *p* < 0.01, ^###^ *p* < 0.001 and ^####^ *p* < 0.0001, as compared to the group in the absence of deS/deGal IgA, respectively. (*n* = 6).

To confirm the effects of tetrandrine, we also examined the effects of tetrandrine on the proliferation of HRMCs. deS/deGal IgA significantly stimulated the proliferation of HRMCs (*p* < 0.05, Figure 5A). Tetrandrine inhibited the proliferation of HRMCs stimulated by deS/deGal IgA dose-dependently and time-dependently with IC_50_ value of 3.16 ± 0.10 μM (48 h), 2.42 ± 0.09 μM (72 h) and 2.13 ± 0.24 μM (96 h) (Figure 5B). 1–3 μM of tetrandrine decreased the percentage of viable cells dose-dependently and significantly on HRMCs with non-stimulation of deS/deGal IgA (*p* < 0.001, Figure 5C). Compared with non-stimulation of deS/deGal IgA, 1–3 μM of tetrandrine showed stronger inhibitory effect on the proliferation of HRMCs with the stimulation of deS/deGal IgA (*p* < 0.05, Figure 5C).

### 3.6 Effects of tetrandrine on the expression of IgA receptor in HRMCs

**FIGURE 6:**
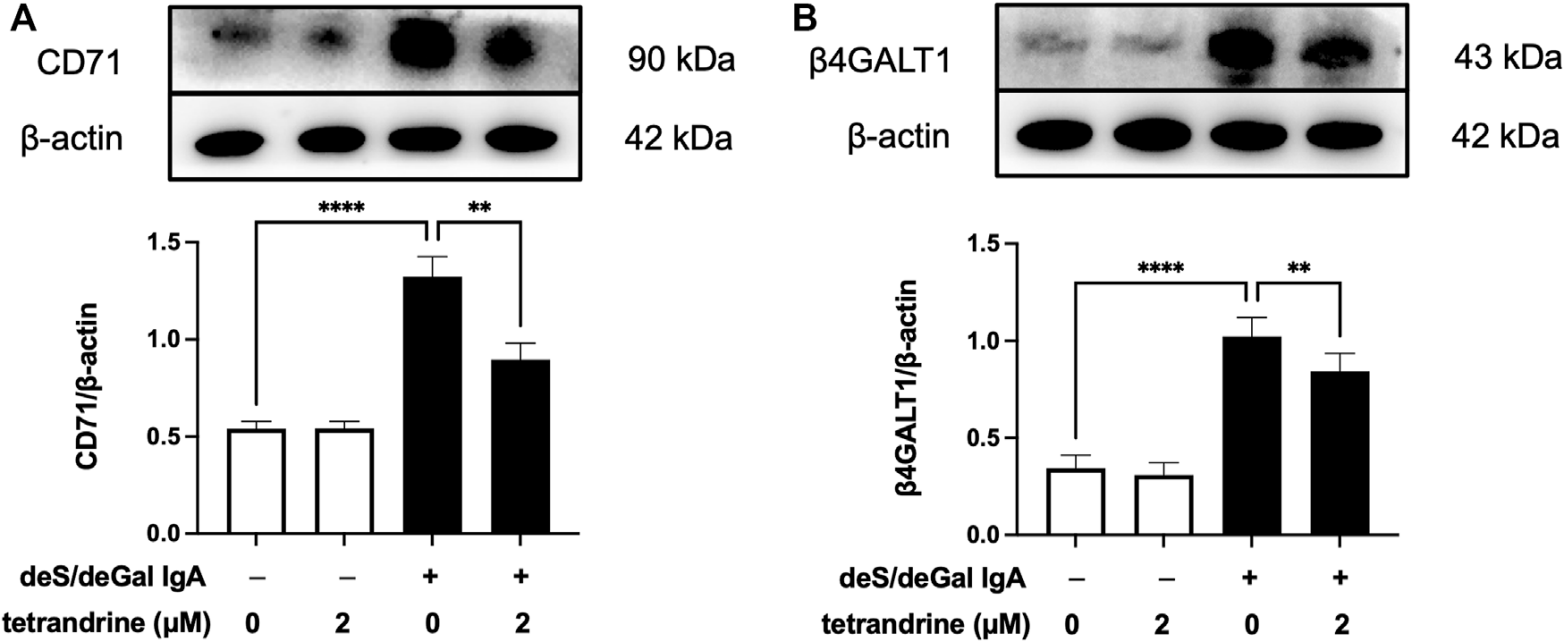
Effects of tetrandrine on the expression levels of IgA receptor protein in HRMCs. Cells were treated with 2 μM of tetrandrine in the presence or absence of deS/deGal IgA for 48 h. The cell lysates were examined by Western blot. More than three independent experiments were carried out and representative results were shown in the figures. *β*-actin was used as internal control. Statistical analyses were performed using Bonferroni’s multiple comparison tests, **p < 0.01 and ****p < 0.0001 respectively. (*n* = 6).

IgA receptor CD71 and β4GALT1 were largely upregulated significantly with the stimulation of deS/deGal IgA (*p* < 0.001, Figure 6). 2 μM of tetrandrine significantly decreased the expression of CD71 and β4GALT1 obviously and significantly (*p* < 0.01, Figure 6).

### 3.7 Effects of tetrandrine on MAPK/NF-κB signaling pathway in HRMCs

**FIGURE 7:**
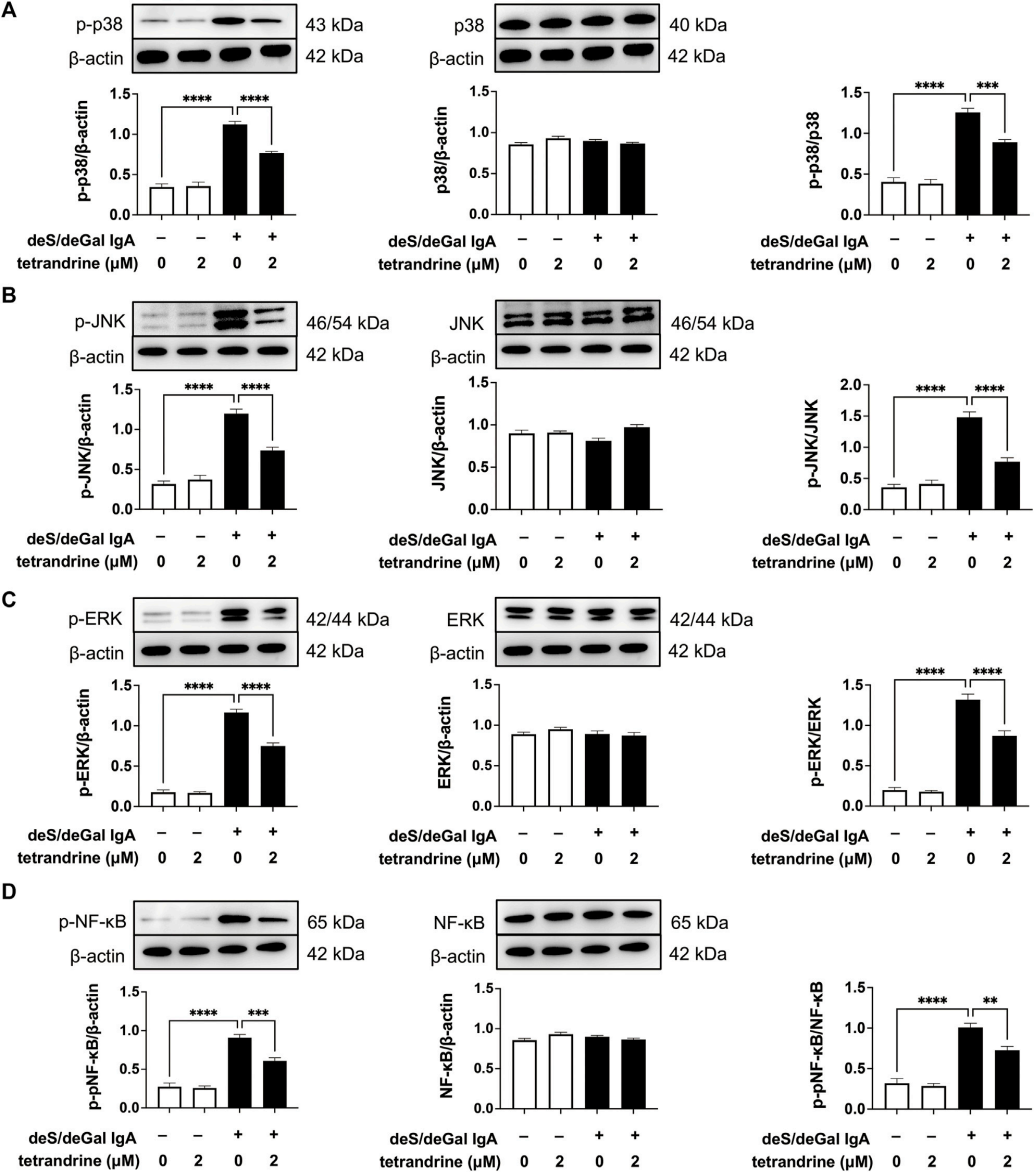
Effects of tetrandrine on the expression levels of MAPK/NF-κB in HRMCs. Cells were treated with 2 μM of tetrandrine in the presence or absence of deS/deGal IgA for 48 h. The cell lysates were examined by Western blot. More than three independent experiments were carried out and representative results were shown in the figures. *β*-actin was used as internal control and re-used to compare the expression levels of MAPK/NF-κB and their phosphorylation protein conveniently. Statistical analyses were performed using Bonferroni’s multiple comparison tests, **p < 0.01, ***p < 0.001 and ****p < 0.0001 respectively. (*n* = 6).

As shown in Figure 7, treatment of deS/deGal IgA significantly stimulated the phosphorylation of p38, JNK, ERK and NF-κB (*p* < 0.001). 2 μM of tetrandrine decreased the expression of p-p38, p-JNK, p-ERK and p-NF-κB significantly with little effect on the expression of p38, JNK, ERK and NF-κB (*p* < 0.001, Figure 7).

## 4 Discussion

Renin-angiotensin system blockade and immunosuppressive drugs, including corticosteroids have been list in the guideline for the therapy of IgAN (Rasche et al., 2016). Although the use of these drugs was confirmed to reduce the risk of kidney failure and slow down the progression of renal function in patients with high-risk IgAN, a large proportion of patient was still observed to develop end-stage kidney disease (Lv et al., 2017; O’Shaughnessy and Lafayette, 2017). An “multi-hit” hypothesis of genetic and molecular mechanisms underlying IgAN is undisputed. Firstly, the production of a typical galactose-deficient mucosal-type IgA1 antibodies increased in peripheral blood circulatory system. Secondly, anti-IgA1 autoantibodies formed in immunological system. Thirdly, IgA1-containing immune complexes deposited in the glomerular mesangium, which incited nephritogenic inflammatory response (O’Shaughnessy and Lafayette, 2017). The immune deposition leads to mesangial cell proliferate and over-produce components of extracellular matrix, cytokines and chemokines, causing downstream podocyte injury and induce proteinuria (Lai et al., 2016). Several studies revealed that tetrandrine had therapeutic potential in reducing proteinuria, improving renal function, and alleviating renal pathological damage via attenuating podocyte injury (Yu et al., 2020; Ding et al., 2021; Mao et al., 2022). To the best of our knowledge, the current research firstly suggested that tetrandrine inhibited the proliferation of mesangial cells induced by enzymatically deglycosylated human IgA1 via IgA receptor/MAPK/NF-κB signaling pathway.

Mesangial deposition of aberrantly glycosylated IgA1 is considered as the key pathogenic process of IgA nephropathy (Wang et al., 2016). The IgA1 hinge region has up to six clustered O-glycans which consist of serine and threonine-linked N-acetylgalactosamine usually with β1,3-linked galactose and variable sialylation (Ohyama et al., 2021). When human IgA1 were enzymatically desialylated or further degalactosylated using neuraminidase and *β*-galactosidase, deglycosylated human IgA1 accumulated in rat glomeruli after injecting into the left renal artery (Sano et al., 2002). In the present study, deS/deGal IgA significantly stimulated the proliferation of both HBZY-1 cells and HRMCs, whereas native IgA and deS IgA showed limited stimulation effects (Figures 1A, 5A). This observation suggested that galactose-deficiency in IgA1 was essential for stimulation function of the deposited IgA1 within the mesangial areas. The stimulation function of galactose-deficiency IgA1 resulted in mesangial cell proliferation, which could be inhibited by the treatment of tetrandrine dose-dependently and time-dependently (Figures 1B, 5B). Compared with non-stimulation of deS/deGal IgA, 1–3 μM of tetrandrine showed stronger inhibitory effect on the proliferation of HRMCs with the stimulation of deS/deGal IgA (*p* < 0.05, Figures 1C, 5C), which suggested that tetrandrine might specifically inhibit the stimulation of deS/deGal IgA.

Pathogenetic IgA only deposit in the mesangial areas of the glomerulus, whereas deposits are rare to find in podocytes or renal tubular epithelial cells, which suggests that IgA receptors are responsible for mesangial cell proliferation induced by galactose-deficiency IgA1 (Lai et al., 2016). CD71 has been identified as a candidate IgA1 receptor expressed on HRMCs. CD71 binds IgA1 and co-localizes with mesangial IgA1 deposits, and is overexpressed in patients with IgAN (Moura et al., 2004). CD71-specific antibodies and transferrin were observed to inhibit the binding of IgA to HRMC partially, suggesting that HRMC expresses at least more than two kinds of IgA receptor. Recently, β4GALT1 was confirmed as an important IgA receptor in HRMC since it played a significant role in the clearance of mesangial IgA and the initial response to IgA deposition (Molyneux et al., 2017). As shown in Figures 2, 6, deS/deGal IgA significantly stimulated the expression of both CD71 and β4GALT1 (*p* < 0.01) in both rat mesangial cell HBZY-1 and human mesangial cell HRMC. 1–3 μM of tetrandrine were observed to downregulate the expression of both CD71 and β4GALT1 obviously, which might contribute to the specific inhibitory effect of tetrandrine against the proliferation of mesangial cell induced by deS/deGal IgA as mentioned in Figures 1C, 5C.

MAPK regulates a variety of cellular processes by transmitting extracellular signals to intracellular reactions and plays an important role in cell proliferation, differentiation, motility, and survival (Cargnello and Roux, 2011). As the common downstream targets, NF-κB family transcription factors are inducible by many different cell-surface receptors, which also regulate cell proliferation, differentiation and death (Xu et al., 2021). Leung et al. reported that polymeric anionic IgA isolated from IgAN patients’ venous blood stimulated the activation of MAPK/NF-κB (Leung et al., 2008). Our study also confirmed that enzymatically deglycosylated human IgA1 induced the phosphorylation of p38, JNK, ERK and NF-κB (*p* < 0.01, Figures 3, 7), which might result in the mesangial cell proliferation as shown in Figures 1A, 5A. In the present study, tetrandrine was observed to inhibit the activation of MAPK/NF-κB (Figures 3, 7), thus, inhibit the proliferation of both HBZY-1 cells and HRMC stimulated by deS/deGal IgA (Figures 1, 5). Additionally, these inhibitory effect of tetrandrine caused cell cycle arrest and stopped the cell growth in the S phase companied with the upregulating of cyclin A2 and downregulating of cyclin D1 in HBZY-1 cells (Figure 4).

## 5 Conclusion

**FIGURE 8:**
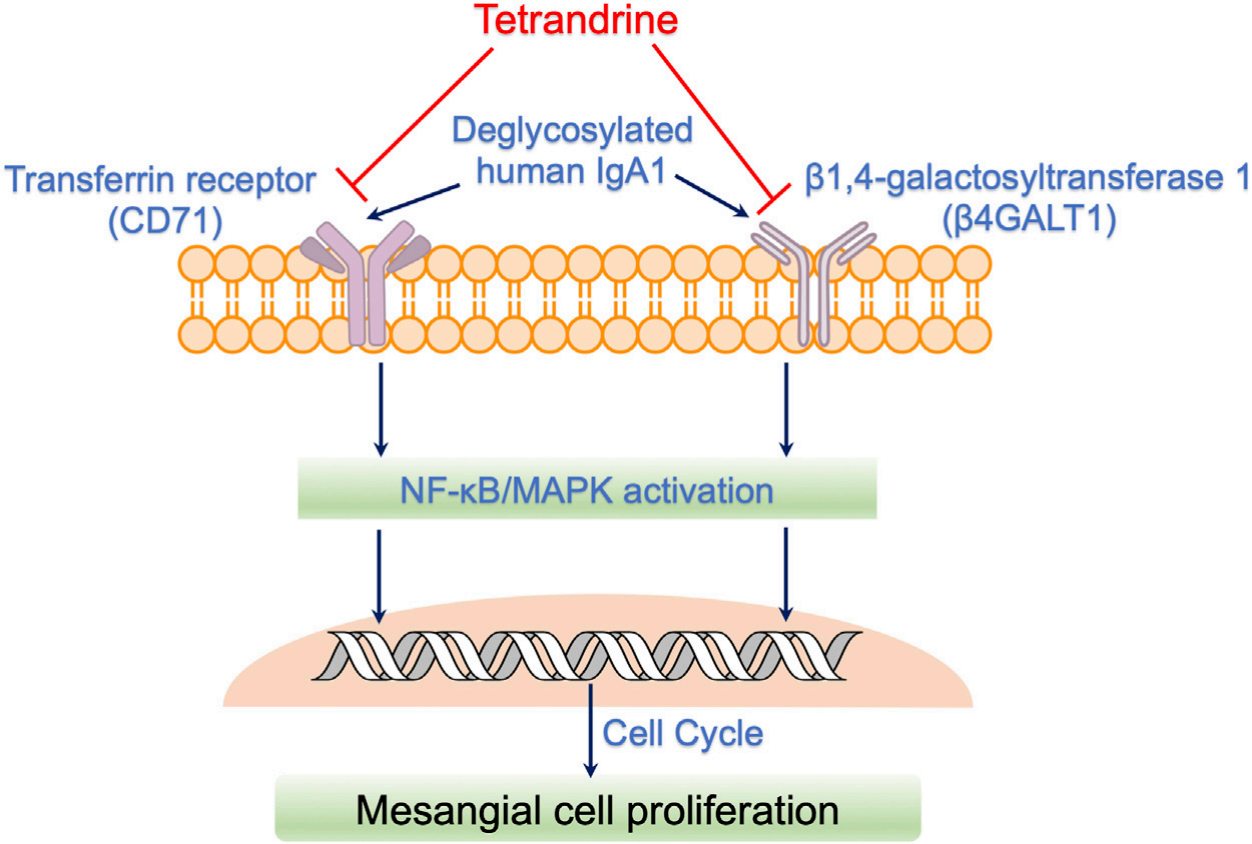
Possible action mechanisms of tetrandrine to inhibit the proliferation of mesangial cells induced by deglycosylated human IgA1. Deglycosylated human IgA1 molecule stimulated the expression of IgA1 receptor, CD71 and β4GALT1. Consequently, MAPK/NF-κB signaling pathway was activated, resulting in triggering cell cycle to regulate mesangial cell proliferation. Tetrandrine decreased the expression of IgA1 receptor, CD71 and β4GALT1, and thus, inhibited the activation of MAPK/NF-κB, resulting in triggering cell cycle arrest to stop at S phase companied with the upregulating of cyclin A2 and downregulating of cyclin D. Taken together, tetrandrine possibly inhibited the proliferation of mesangial cells induced by enzymatically deglycosylated human IgA1 via IgA receptor/MAPK/NF-κB signaling pathway.

In conclusion, enzymatically deglycosylated human IgA1 molecule stimulated the proliferation of both rat mesangial cell HBZY-1 and human mesangial cell HRMC. Compared with non-stimulation of deS/deGal IgA, 1–3 μM of tetrandrine showed stronger inhibitory effect on the proliferation of HBZY-1 cells and HRMCs with the stimulation of deS/deGal IgA (*p* < 0.05), suggesting that tetrandrine possibly inhibited the proliferation of mesangial cells induced by deglycosylated human IgA1 specifically. Molecular mechanism study revealed that tetrandrine decreased the expression of IgA1 receptor, CD71 and β4GALT1, and inhibited the activation of MAPK/NF-κB, resulting in triggering cell cycle arrest to stop at S phase companied with the upregulating of cyclin A2 and downregulating of cyclin D1. Taken together, tetrandrine inhibited the proliferation of mesangial cells induced by enzymatically deglycosylated human IgA1 via IgA receptor/MAPK/NF-κB signaling pathway (Figure 8). Based on these potential molecular mechanisms, tetrandrine would be an appealing therapeutic option for IgAN.

## Data Availability

The original contributions presented in the study are included in the article/supplementary material, further inquiries can be directed to the corresponding authors.
